# *MAVS* is not a Likely Susceptibility Locus for Addison’s Disease and Type 1 Diabetes

**DOI:** 10.1007/s00005-016-0426-6

**Published:** 2016-09-20

**Authors:** Magdalena Zurawek, Marta Fichna, Marta Kazimierska, Piotr Fichna, Agnieszka Dzikiewicz-Krawczyk, Grzegorz Przybylski, Marek Ruchala, Jerzy Nowak

**Affiliations:** 1 Institute of Human Genetics, Polish Academy of Sciences, Strzeszynska 32, 60-479 Poznan, Poland; 2 Department of Endocrinology and Metabolism, Poznan University of Medical Sciences, Poznan, Poland; 3 Department of Paediatric Diabetes and Obesity, Poznan University of Medical Sciences, Poznan, Poland

**Keywords:** Addison’s disease, Type 1 diabetes, *MAVS*, Variants

## Abstract

Mitochondrial antiviral signaling (MAVS) protein is an intracellular adaptor molecule, downstream of viral sensors, retinoid acid-inducible gene I (RIG-I)-like receptors (RLRs). Impaired antiviral cell signaling might contribute to autoimmunity. Studies have recently shown variations in genes encoding RLRs as risk factors for autoimmune diseases. We investigated whether *MAVS* coding polymorphisms are associated with Addison’s disease (AD) and type 1 diabetes (T1D) in Polish population. We genotyped 140 AD, 532 T1D patients and 600 healthy controls for *MAVS* rs17857295, rs7262903, rs45437096 and rs7269320. Genotyping was performed by TaqMan assays. Distribution of the *MAVS* genotypes and alleles did not reveal significant differences between patients and controls (*p* > 0.05). This analysis did not indicate the association of the *MAVS* locus with susceptibility to AD and T1D.

## Introduction

Addison’s disease (AD) and type 1 diabetes (T1D) are autoimmune endocrine disorders, characterized by T cell mediated progressive destruction of adrenal cortex and pancreatic beta cells, respectively. The pathogenesis of AD and T1D is a consequence of complex process, influenced by a combination of genetic and environmental factors. Epidemiological and research studies designate viral infections as plausible environmental triggers for autoimmunity in genetically susceptible individuals. Viral molecules are recognized by cytoplasmic retinoic acid-inducible gene I (RIG-I)-like receptors: melanoma differentiation-associated factor 5 (MDA5), RIG-I and laboratory of genetics and physiology 2. RIG-I-like receptors as well as Toll-like receptors (TLRs), are members of pattern recognition receptors (PRRs) and have emerged as key sensors of viral infection (Thompson and Locarnini 2007). RLRs detect RNA derived from ssRNA-viruses (*Orthomyxoviridae*, *Paramyxoviridae*, *Rhabdoviridae*, *Picornaviridae, Coronaviridae*), dsRNA-viruses (*Reoviridae*) and in some instances dsDNA-viruses (Epstein-Barr virus, Herpes simplex virus-1 and Adenovirus) (Dixit and Kagan 2013).

Upon activation, RIG-I-like receptors (RLRs) recruit antiviral mitochondrial transmembrane protein MAVS, also known as Cardif, VISA and IPS-1, resulting in downstream signaling cascade of transcription factors and type I interferon (IFN) production.

Aberrant stimulation of RLRs signaling pathway might lead to autoimmunity. Mutations in MDA5 have been reported in patients with systemic lupus erythematosus (SLE) (Van Eyck et al. 2015) and autoinflammatory disorders: Aicardi–Goutieres syndrome (Oda et al. 2014; Rice et al. 2014) and Singleton–Merten syndrome (SMS) (Rutsch et al. 2015). Also, mice with MDA5 missense mutation (G821S) spontaneously develop lupus-like symptoms (Funabiki et al. 2014). The genome-wide association studies and independent association analyses have indicated the common and rare variants in *IFIH1* gene, encoding MDA5, as risk factors in autoimmune disease (Barrett et al. 2009; Gateva et al. 2009; Genetic Analysis of Psoriasis et al. 2010; Reddy et al. 2011). Jang et al. (2015) demonstrated that mutations of *DDX58*, whose protein product is RIG-I, cause the atypical SMS.

Moreover, recent findings indicated an association between single nucleotide polymorphisms (SNPs) or copy number variations (CNVs) in TLRs and the onset of several autoimmune conditions, such as T1D (Broen et al. 2012; Sun et al. 2014), rheumatoid arthritis (Davis et al. 2015), SLE (Enevold et al. 2014; Pacheco et al. 2014) and increased susceptibility to autoimmunity (Nahum et al. 2011).

The evidence of abnormal activation of PRRs-dependent signaling in autoimmune disease, affected by CNVs and SNPs, prompted us to investigate whether variants of *MAVS* gene might influence the risk for AD and T1D in Polish population.

## Materials and Methods

### Study Populations

The association study was performed in a cohort of 140 AD (101 females and 39 males), 532 T1D patients (275 females and 257 males) and 600 healthy control subjects (372 females and 228 males) from the local, homogenous Polish population of Caucasian origin. Clinical diagnosis of AD was confirmed by either low basal serum cortisol with a high adrenocorticotropin level, or subnormal response to short synacthen test (Husebye et al. 2014). The diagnosis of T1D was based upon World Health Organization criteria and all patients required insulin therapy. Autoimmune etiology of AD was confirmed by positive serum autoantibody to adrenal antigens. In the past, these antibodies had been evaluated by an in-house solid-phase radioimmunoassay using microsomal fraction of human adrenals, whereas in 116 more recently diagnosed individuals—by a commercial radioimmunoassay specifically detecting autoantibodies to 21-hydroxylase (RSR Ltd, Cardiff, UK). At diagnosis, patients with T1D were tested for autoantibodies to insulin (positive in 44.5 %), glutamic acid decarboxylase (positive in 66.3 %) and islet antigen IA2 (positive in 72.4 %) using commercially available radioimmune assays (Diasource KIP0091, Euroimmun RA 1022-10001 and RA 1023-10001, respectively). Mean age at disease onset was 32.6 (±10.9) and 8.5 (±4.4) years for AD and T1D, respectively. Additionally, 119 (85.0 %) patients with AD and 79 (14.8 %) individuals with T1D presented concomitant autoimmune disorders, most frequently autoimmune thyroid disease (Table 1). Control samples were obtained from healthy blood donors with negative history of autoimmunity and no signs of autoimmune disorders, recruited at the Regional Blood Transfusion Centre. Their mean age was 38.1 (±10.3) years. This research was approved by local ethics committee and informed consent was obtained from study participants.

**Table 1:** Coexisting autoimmune disorders in studied patients with Addison’s disease (AD) and type 1 diabetes (T1D)

	AD *n* = 140 (%)	T1D *n* = 532 (%)
AITD	105 (75.0)	70 (13.2)
Hashimoto’s thyroiditis	81 (57.9)	64 (12.0)
Graves’ disease	24 (17.1)	6 (1.1)
Chronic atrophic gastritis ± pernicious anemia	21 (15.0)	3 (0.6)
T1D	14 (10.0)	NA
Celiac disease	2 (1.4)	4 (0.8)
Hypergonadotropic hypogonadism	9 (6.4)	–
Vitiligo	8 (5.7)	2 (0.4)
Alopecia	3 (2.1)	2 (0.4)
Myasthenia gravis	–	1 (0.2)

*AITD* autoimmune thyroid disease

### Genotyping

Coding variants of *MAVS* gene (rs17857295, rs7262903, rs45437096, rs7269320) were selected for genotyping according to the previous association studies (Liu et al. 2011; Varzari et al. 2014). Genomic DNA was extracted from peripheral blood using Puregene Blood Core Kit C (QIAGEN Sciences, USA). Genotyping was performed using TaqMan assays (C_25749920_10, C_86397448_10, C_25623845_10, C_25623847_10, Applied Biosystems by Thermo Fisher Scientific, USA) in Bio-Rad CFX96 Real-Time Detection System. Genotypes of all studied polymorphisms were confirmed by direct DNA sequencing of both strands by BigDye^®^ Terminator v3.1 and 3730xl Genetic Analyzer (Thermo Fisher Scientific, USA). The controls of confirmed genotypes were used in all genotyping reactions.

### Statistical and Bioinformatics Analysis

Hardy–Weinberg equilibrium was tested for each studied single nucleotide polymorphisms in AD, T1D patients and controls using χ^2^ test Court Lab HW calculator available online (www.tufts.edu). χ^2^ test (or Fisher’s exact test when appropriate) on 2 × 3 and 2 × 2 contingency tables was applied to assess differences in genotype distribution and allele frequencies, respectively. These calculations were performed using GraphPad Prism 5 (GraphPad Software, USA). Linkage disequilibrium (LD) measures (Lewontin’s D’) between *MAVS* variants were calculated using Haploview version 4.2 available online (http://www.broadinstitute.org). Haplotype frequencies were compared among patients and controls using Chi^2^ test. The power to detect an association with disease at the 0.05 level of significance was calculated with online PS Power and Sample Size calculator 3.1.2 (http://biostat.mc.vanderbilt.edu/wiki/Main/PowerSampleSize). The functional context of nonsynonymous variants rs17857295, rs7262903, rs45437096 and rs7269320 was predicted using Protein Variation Effect Analyzer (PROVEAN, http://provean.jcvi.org/index.php). The variants with PROVEAN cutoff score below −2.5 were pinpointed as a deleterious.

### Results and Discussion

All studied *MAVS* variants were in Hardy–Weinberg equilibrium in AD, T1D and control group (*p* > 0.05). The frequencies of alleles and genotypes of the rs17857295, rs7262903, rs45437096, rs7269320 did not present significant differences between AD, T1D patients and healthy controls (Table 2). The power calculation, assuming an allelic odds ratio 1.5 and given the minor allele frequency (MAF) 24, 14.5, 17, 14.3 % of rs17857295, rs7262903, rs45437096, rs7269320, respectively, yielded 65 % (MAF 14.3 %)–80 % (MAF 24 %) in AD and 95 % (MAF 14.3 %)–99 % (MAF 24 %) in T1D group.

**Table 2:** Distribution of the *MAVS* polymorphisms in patients with Addison’s disease (AD), type 1 diabetes (T1D) and healthy controls (CON)

SNP	Amino acid change	Genotype	Allele	AD *n* = 140 (%)	*p*	T1D *n* = 532 (%)	*p*	CON *n* = 600 (%)
rs17857295 C/G	Gln93Glu	CC		74 (52.8)		293 (55.1)		348 (58.0)
		CG		61 (43.6)		207 (38.9)		213 (35.5)
		GG		5 (3.6)	0.12	32 (6.0)	0.49	39 (6.5)
			C	209 (74.6)		793 (74.5)		909 (76.0)
			G	71 (25.4)	0.70	271 (25.5)	0.50	291 (24.0)
rs7262903 C/A	Gln198Lys^a^	CC		97 (69.3)		389 (73.1)		438 (73.0)
	Gln57Lys^b^	CA		40 (28.6)		133 (25.0)		150 (25.0)
		AA		3 (2.1)	0.67	10 (1.9)	0.99	12 (2.0)
			C	234 (83.6)		911 (85.6)		1026 (85.5)
			A	46 (16.4)	0.41	153 (14.4)	0.93	174 (14.5)
rs45437096 C/T	Arg218Cys^a^	CC		95 (67.9)		361 (67.9)		417 (69.5)
	Arg77Cys^b^	CT		42 (30.0)		151 (28.4)		165 (27.5)
		TT		3 (2.1)	0.74	20 (3.7)	0.71	18 (3.0)
			C	232 (82.9)		874 (82.1)		999 (83.0)
			T	48 (17.1)	0.87	190 (17.9)	0.49	201 (17.0)
rs7269320 C/T	Ser409Phe^a^	CC		98 (70.0)		392 (73.7)		440 (73.3)
	Ser268Phe^b^	CT		40 (28.6)		135 (25.4)		148 (24.7)
		TT		2 (1.4)	0.59	5 (0.9)	0.34	12 (2.0)
			C	236 (84.3)		919 (86.4)		1028 (85.7)
			T	44 (15.7)	0.56	145 (13.6)	0.63	172 (14.3)

*p* values refer to comparisons with the controls (Chi^2^ or Fisher’s exact tests)

Position of amino acid change in MAVS protein ^a^ isoform 1, ^b^ isoform 2

Linkage disequilibrium measurement revealed strong LD between rs7262903, rs45437096 and rs7269320 in AD, T1D and control group with Lewontin’s *D*’ values ranging between 0.89 and 0.99. Analysis of haplotypes including rs7262903, rs45437096, rs7269320 did not show statistically significant differences between AD, T1D cohorts and healthy controls (*p* > 0.05; data not shown). The PROVEAN software predicted rs7262903 (Gln57Lys), rs45437096 (Arg77Cys) and rs7269320 (Ser268Phe) of MAVS protein, isoform 2 as deleterious with PROVEAN scores −5.07, −4.29, −3.49, respectively.

Current study did not reveal an association between *MAVS* variants and AD and T1D. In line, association analysis in German multiple sclerosis (MS) patients (Varzari et al. 2014) and in SLE patients in Chinese population (Liu et al. 2011) did not provide robust evidence that *MAVS* polymorphisms might be risk factors for autoimmune diseases. Evaluation of variants rs4815617, rs17857295, rs7262903, rs45437096 and rs16989000 did not indicate any significant single-SNP association with MS risk, only a modest effect on age at disease onset was observed for rs4815617 and 7262903 (Varzari et al. 2014). However, Varzari et al. (2014) showed evidence for combinatorial effect of polymorphic *MAVS* variants rs45437096 and rs10813825, rs12985 in *RIG*-*I* and *ACO1* gene (Aconitase 1) on MS risk. The association study of *MAVS* rs17857295, rs2326369, rs7262903 and rs7269320 in a cohort of Chinese patients with SLE did not reveal any SNP as a risk factor for systemic lupus erythematosus (Liu et al. 2011).

Our in silico analysis of variants tested in the current study with PROVEAN software tool predicted a functional implication for rs7262903, rs45437096 and rs7269320. However, the molecular screening of *MAVS* coding variants in human cell lines did not pinpoint rs17857295, rs7262903, rs45437096 and rs7269320 to have an impact on the biological function of the protein (Pothlichet et al. 2011). The polymorphisms investigated in the current study for an association with AD and T1D seem to be neutral rather than protein disruptive. On the contrary, Pothlichet et al. (2011) demonstrated rs11905552 (Cys79Phe) as a critical loss-of-function variant in the *MAVS* gene. This SNP reduced expression of type I IFN and other proinflammatory mediators in vitro and was associated with low type I IFN production and absence of anti-RNA-binding protein autoantibodies in African–American SLE patients (Pothlichet et al. 2011).

In conclusion, *MAVS* variants are not likely to be associated with the risk for AD and T1D in Polish population. However, accumulating evidence of interactions between innate immune system and autoimmunity, require further research focusing on the molecular characterization of RLRs signaling pathway in autoimmune conditions.
